# Unacknowledged Pain and Disenfranchised Grief: A Narrative Analysis of Physical and Emotional Pain in Complex MAiD Bereavement Stories

**DOI:** 10.1080/24740527.2023.2231046

**Published:** 2023-08-15

**Authors:** Kristie Serota, Michael Atkinson, Daniel Z Buchman

**Affiliations:** a Dalla Lana School of Public Health, University of Toronto, Toronto, Ontario, Canada; b Kinesiology and Physical Education, University of Toronto, Toronto, Ontario, Canada; c Centre for Addiction and Mental Health (CAMH), Toronto, Ontario, Canada; d Joint Centre for Bioethics, University of Toronto, Toronto, Ontario, Canada

**Keywords:** Medical assistance in dying, emotional pain, bereavement, family, disenfranchised grief

## Abstract

**Background:**

Pain can influence an individual’s choice to pursue medical assistance in dying (MAiD) and may also influence how family members experience that decision. Family conflict or discordance surrounding a loved one’s MAiD decision can cause unique challenges affecting grief and bereavement, including disenfranchised grief. There is limited knowledge of how individuals with complex MAiD bereavement experiences describe the role of physical and emotional pain in their bereavement stories.

**Aims:**

This article explores the role of physical and emotional pain in the stories of family members with complex MAiD bereavement and identifies opportunities to improve care for individuals and families experiencing disagreement around MAiD.

**Methods:**

We conducted qualitative interviews and utilized a narrative and ethics of care approach to analyze the data.

**Results:**

We conducted *N* = 12 narrative interviews with participants in three provinces: Ontario, British Columbia, and Alberta. Descriptions of physical pain were used to justify the morality, or immorality, of MAiD in the context of patient suffering. Emotional pain described experiences where participants’ feelings about MAiD went unacknowledged by their family or friends, institutions, and sociopolitical environments. We conceptualize this unacknowledged emotional pain as disenfranchised grief and make recommendations to improve care for individuals experiencing complex MAiD bereavement.

**Conclusions:**

Experiences of physical and emotional pain leave a lasting impact on family members with complex MAiD bereavement. Health care professionals should continue to improve care for family members following MAiD, especially where there is disagreement or family conflict.

## RÉSUMÉ

**Contexte:** La douleur peut influencer le choix d’une personne de demander l’aide médicale à mourir (AMM) et peut également influencer la façon dont les membres de la famille vivent cette décision. Le conflit ou la discorde au sein de la famille entourant la décision d’un être cher d’avoir recours à l’AMM peut entrainer des difficultés en ce qui concerne le chagrin et au deuil, notamment le deuil privé de ses droits. La façon dont les individus vivant l’expérience complexe du deuil lié à l’AMM décrivent le rôle de la douleur physique et émotionnelle dans leur histoires de deuil est peu connue.

**Objectifs:** Cet article se penche sur le rôle de la douleur physique et émotionnelle dans les histoires des membres de la famille vivant un deuil complexe lié à l’AMM et décrit les occasions d’améliorer les soins destinés aux individus et aux familles connaissant un désaccord autour de l’AMM.

**Méthodes:** Nous avons mené des entretiens qualitatifs et utilisé une approche narrative axée sur l’éthique des soins pour analyser les données.

**Résultats:** Nous avons mené N = 12 entretiens narratifs avec des participants de trois provinces : l’Ontario, la Colombie-Britannique et l’Alberta. Des descriptions de la douleur physique ont été utilisées pour justifier la moralité, ou l’immoralité, de l’AMM dans le cadre de la souffrance du patient. La douleur émotionnelle décrit l’expérience oo les sentiments des participants au sujet de l’AMM qui n’ont pas été reconnus par leur famille ou leurs amis, les institutions et l’environnement sociopolitique. Nous conceptualisons cette douleur émotionnelle non reconnue comme le deuil privé de ses droits et faisons des recommandations pour améliorer les soins pour les personnes qui vivent un deuil complexe lié à l’AMM.

**Conclusions:** L’expérience de la douleur physique et émotionnelle a un effet durable sur les membres de la famille qui vivent un deuil complexe en lien avec l’AMM. Les professionnels de la santé devraient continuer à améliorer les soins destinés aux membres de la famille après l’AMM, en particulier en cas de désaccord ou de conflit familial.

[Audibly crying] For the first time in months, all these pain lines on his face disappeared. He was finally relaxed. It was—it was tough to watch, but that’s what he—it was nice to see him finally not in pain. (Evan)

## Background

In 2016, Bill C-14^1^ legalized a process in which eligible adults can request that a physician or nurse practitioner provide lethal medication to end their lives. To be eligible, individuals must have a grievous and irremediable medical condition, be in an advanced state of decline, and experience intolerable suffering. In 2021, Bill C-7^2^ created a two-track approach to medical assistance in dying (MAiD) based on whether death is reasonably foreseeable.

Inadequate control of pain, or concerns about controlling pain, is the third most common source of suffering noted in MAiD assessments.^3^ Pain can influence an individual’s choice to pursue MAiD and may also influence how that decision is experienced by family members.^4,5^ Improving understanding of how physical and emotional pain affect family members may illuminate opportunities for improving the resources and supports available during and after the MAiD process.

Family members^a^^a^We take a broad definition of family, including the family of origin, chosen family, and close friends. have no formal role in the MAiD process. Research suggests that many family members support MAiD, and their bereavement may be no more challenging than that following natural death.^4,6–9^ However, other studies concluded that MAiD bereavement has unique and challenging features, including navigating policy requirements, scheduling the date, interacting with clinicians, experiencing stigma, and disclosing the cause of death to others.^10–15^ Those who experience discordance or family conflict over the MAiD decision report greater challenges with grief and bereavement.^10,15,16^

### Emotional Pain and Disenfranchised Grief

Some family members who disagree about MAiD may experience emotional pain. Timulak described emotional pain as a challenging, overwhelming internal experience that is the “response to an [emotional] injury that prevents or violates the fulfillment of the basic human needs, such as being loved, safe, and acknowledged.”^17(p5)^ Emotional pain may include psychological and physiological symptoms, including anxiety, panic attacks, depression, obsessive thoughts, insomnia, change in appetite, and muscular tension.^17–19^

If an individual’s need to be acknowledged is undermined in the context of MAiD disagreement, the resulting emotional pain can be conceptualized as disenfranchised grief.^17^ Doka described disenfranchised grief as “the grief experienced by those who incur a loss that is not, or cannot be, openly acknowledged, publicly mourned or socially supported.”^20(p37)^ Deaths by suicide or drug overdose are examples of deaths that are disenfranchised and may lead to stigma and isolation. Assisted dying has also been classified as disenfranchised following this definition.^11,15^

Individuals with negative MAiD experiences or disenfranchised grief may be less likely to participate in research studies.^5,8,13^ We thus developed the current study to explore the complex MAiD bereavement experiences of family members who had disagreements, family conflicts, or discordant views on MAiD. This article explores the role of physical and emotional pain in family members’ stories and identifies how care may be improved for those with complex MAiD experiences.

## Methods

### Ethics

The University of Toronto Research Ethics Board (No. 41677) approved this study. All participants signed a consent form and were assigned a pseudonym. The data have been de-identified to protect the confidentiality of participants.

### Recruitment and Sample

Participants were recruited through two newsletters from community organizations, Canadian Virtual Hospice and Bridge C-14; social media, Twitter, and Facebook; one professional organization, The Canadian Association of MAiD Assessors and Providers; two therapists who asked whether they could share our information with their clients; and snowball sampling. We were contacted by 21 individuals who were interested in participating in the study. After screening for eligibility, 15 were invited to participate, and 12 chose to complete a narrative interview.

All participants were at least 18 years old; were able to complete an interview in English; had a family member or close friend who had died by MAiD at least 4 months prior; experienced some disagreement, conflict, or discordant views about the MAiD decision; and were not currently undergoing psychological treatment or counseling to manage the grief associated with this loss. Individuals currently accessing counseling were excluded from participating in the study to avoid interfering with their treatment. However, individuals who had finished treatment were eligible to participate.

### Data Collection

Before each interview, participants were sent a copy of the interview guide and a list of bereavement resources. Participants chose to complete the interview by telephone (*n* = 5) or Zoom (*n* = 7), and interviews lasted between 41 and 94 min. The narrative interviews began with participants being asked to share their experience with MAiD in their own words. Following a participant’s story presentation, the interviewer used the interview guide to converse about the context and meaning of the narratives.^21,22^ We adapted our interview guide based on Beuthin and colleagues’ study of grief bereavement following a medically assisted death.^6^ All interviews were conducted and transcribed by the lead author, who had experience conducting research interviews on the topic of MAiD.

### Participant Demographics

See Table 1 for participant demographic characteristics.

### Data Analysis

Narrative approaches in empirical bioethics research provide a means for exploring participants’ values and opinions on controversial topics, uncovering meaning-making practices, and exploring the impact of social context.^23^ This approach allowed us to explore family members’ unique experiences through the stories they told about MAiD. The stories are understood to be co-produced between the participant and researcher within the context of the narrative interview.^24^ We conceptualized participants’ stories as social actors, which allowed us to examine “what the story does, rather than understanding the story as a portal into the mind of a storyteller.”^24(p13)^ Through this approach, we identified the pain narratives in participants’ stories and explored how their descriptions of physical and emotional pain were embedded within biomedical knowledge, institutions, and Canada’s MAiD legislation.^22,25^ In the Discussion section, we discuss the ethics of care perspective used to recognize how care is implicated in the power structures that govern social life and suggest emotional pain mitigation strategies to improve care for family members with complex MAiD experiences.^26^ Tronto’s^26^ practice of care ethics requires researchers to be attentive, responsible, competent, and responsive to participants’ experiences and meaning-making practices.

### Coding

We coded the narrative interviews using NVivo 12^27^ to examine how participants articulate different pain formulations in their stories. Pain codes were organized into two categories, descriptions of the patient’s physical pain and participants’ experiences of emotional pain. These codes were then analyzed in the context of participants’ stories. Participants’ emotional pain experiences were further divided into three categories: interpersonal relationships, institutional concerns, and sociopolitical environment.

### Findings

Family members with complex MAiD bereavement experiences use descriptions of pain in their bereavement narratives in two ways. First, physical pain is used to justify the (im)morality of MAiD in the context of their loved one’s suffering. Second, emotional pain described experiences where their feelings about MAiD went unacknowledged in their interpersonal relationships, in institutions, or in sociopolitical environments. We conceptualize this unacknowledged emotional pain as disenfranchised grief.

### Physical Pain in MAiD Bereavement Narratives

Family members used descriptions of physical pain to justify the moral permissibility of MAiD. Vivid images of physical pain portray MAiD as a compassionate choice, even for those who initially felt conflicted by the MAiD decision.

Gloria struggled with her husband’s decision to have MAiD because of her personal religious faith:

I think personally I struggled with his decision in the beginning because of my faith, and I kind of had to get to a place where I could say it’s not my decision, it’s his.

Gloria described the experience of witnessing her husband’s death:

The doctor explained exactly what was going to happen, you know, to the fact that he was going to hiccup, and that would be it, and he would just peacefully go. … I said I love you [long pause], and then he died [crying]. And the interesting thing was, I looked at him and I had kind of an involuntary smile. I think there was a lot of things going on. Relief for him and for me, that he wasn’t suffering any longer, and he wasn’t in pain. And it wasn’t like a happy smile. I think it was just—my priest said, “Your face changed.” And I think it was just that, oh, my goodness, it’s over. The suffering and for him to be at peace, and that was a surprise.

In Gloria’s narrative, pain acts as a moralizing agent. The description of how MAiD alleviated her husband’s pain and suffering justified Gloria’s decision to support her husband’s choice, despite her personal concerns about the moral permissibility of the procedure. MAiD is thus portrayed as a compassionate end-of-life option for those experiencing irremediable suffering. Alleviating pain may be used as moral justification for MAiD by family members who have complex feelings about MAiD’s moral permissibility.

Descriptions about the absence of physical pain also featured in relatives’ MAiD bereavement stories. The perceived absence of physical pain was used to question whether the patient should have met MAiD’s eligibility criteria.

Brianna struggled with her close friend’s decision and worried that she chose MAiD because she felt like a burden:

The last time she raised [MAiD], I said, I don’t think it’s for you because you’re actually healthy. I mean, you have this condition, but you’re not—you’re not dying, it’s not on your doorstep.

She described the experience of witnessing her friend’s MAiD:

There’s something awful about watching that poison. It’s not just peace and wonderful, there’s a disconnect. … Now, if somebody was, you know, in extreme pain, I would say, how fast can it happen? You know. both of my parents died through palliative sedation. They were both very sick with cancer, and they gave them an awful lot of morphine. And they asked for palliative sedation, which basically puts you right under and quite quickly stops your organs from—they close down and you die.

Brianna’s story situates MAiD as only being acceptable for people experiencing extreme pain at the end of life. Because Brianna perceived her friend’s physical pain as being under control, she believed she should not have met the criteria for MAiD. Brianna contrasted her friend’s pain presentation with that of her parents and suggested that if someone’s pain is not extreme enough to receive palliative sedation, they should not be eligible for MAiD. Extreme pain may be used as a precedent for MAiD eligibility by family members with complex feelings about their loved one’s decision.

### Emotional Pain in MAiD Bereavement Narratives

Emotional pain described experiences where participants’ feelings about MAiD went unacknowledged in interpersonal relationships, within institutions, and in sociopolitical environments.

### Emotional Pain Related to Interpersonal Relationships

Family conflict surrounding MAiD can cause emotional pain and complex bereavement.

Faye described her brother’s MAiD death as a traumatic experience. Faye did not think her brother should be eligible for MAiD, and her family was angered by her opposition:

Terrible, absolutely devastating … it was terrible. I couldn’t eat, I couldn’t do anything. I lost a lot of people to cancer and other illnesses, and it was not like this. I mean, it was very hard. For the first week, I think I just—this sounds really silly, but—I just watched home improvement shows on my computer. Like, I’m not interested in home improvement, but I had to do something not in any way related to my life. I couldn’t look at e-mails, I completely left my life somehow. It was really awful. So, I read one magazine, I think a hundred times trying to take my mind off of things. … It was like all I could do, and then I went back to my therapist, who I hadn’t seen in a long time. Not the one that dropped me for whatever reason, but I went back to my former one and was there for two years just dealing with this. So, yeah, that’s what it was like, really hard, and I’m not okay today. I’m still not okay, and I don’t think I ever will be. I’ve never been through such trauma.

Faye’s opposition to MAiD was met with anger from her family members, who supported the decision. Faye’s emotional pain went unacknowledged by her family and the medical institution where the MAiD occurred, in addition to the perceived abandonment by her therapist who “dropped” her. The family was not offered resources to help them navigate their disagreement. Faye joined a suicide survivor’s support group, which helped her process her traumatic grief experience. The MAiD experience can be painful and traumatic for relatives with complex feelings about MAiD, and unacknowledged emotional pain can result in disenfranchised grief.

### Emotional Pain Related to Institutional Concerns

Participants discussed challenging experiences related to institutional or health systems that caused emotional pain. Challenges included negative experiences with clinicians, navigating health systems, accessing resources, and filing complaints when there were concerns about potential wrongdoing.

Ian experienced emotional pain following his father’s MAiD:

In the autumn I started having nightmares, started to wake up at night—I still do about it. I felt sick about it, couldn’t focus on work or anything else like that. … If I would start talking about it, I would start crying, the emotional well-up, that still happens sometimes, but I’m pretty well put together these days.

Ian described his negative experience interacting with the MAiD provider. He detailed his quest for answers about why his father’s MAiD happened as quickly as it did and the various institutions he has sought information from:

I was absolutely appalled and shocked and, like, flabbergasted by how this transpired. The kind of—it just seemed to be cavalier, ignorant—it didn’t seem like anybody ever bothered reading any of the materials my family had sent, or I had sent on behalf of them. It was just like it didn’t matter. I couldn’t understand how not eating and not treating an infection could somehow be construed as an irremediable illness, a terminal illness. … I decide[d] to file a complaint with the College of Physicians and Surgeons [CPS], and so I wrote that up and wrote out a long explanation of his background with all the materials I had and transcripts of the text messages and stuff like this. They wrote back and basically said, if this looks like ineligibility for MAiD, we look at this as a police matter. … So, then a couple months later, I filed a police report. It got as far as major crimes and somebody at major crimes reviewed it within like a day or two and said that, you know, it doesn’t appear to meet threshold for criminal charges, so we’re not going to look into this more. So, the last thing I did was write to the chief coroner and I haven’t heard anything from them. Because there’s something just off about the whole thing. Also, in there, in between what I wrote to the CPS and the police, my mum and I filed to get his medical records around MAiD. … The hospital denied me the full record.

Ian’s concerns about his father’s case were unacknowledged by the MAiD provider, the hospital, the college, and the police. He continues to wait for a response from the chief coroner. Despite his various concerns, none of the institutions he has spoken to has yet to validate his concerns. This lack of acknowledgment at the institutional level results in disenfranchised grief. Like Faye, Ian sought grief support through a suicide survivor’s support group.

### Emotional Pain Related to the sociopolitical Environment

Participants with chronic illness and disabilities spoke about the challenges of seeing their close friends access MAiD in a social and political climate where health care can be inaccessible to people with disabilities. In this final section, we discuss the emotional pain of feeling marginalized in contemporary Canadian society.

Hollis described their experience seeking support following their close friend’s MAiD death. Their friend was described as living in extreme poverty, receiving disability benefits, and living in unsafe housing.

People have tried to help me by offering me grief resources and stuff, and it’s like none of these apply to me because I genuinely feel like I watched my friend get murdered. Like, that is my experience of this situation. … ’Cause it wasn’t an empowered choice. She was really clear about why she made that decision, and it wasn’t because of her pain, it wasn’t because of her illness, it wasn’t because of her disabilities … she did this because she didn’t have the things she needed to live a good life as a disabled person. And it’s impossible to heal from something like this when it’s not over, because it isn’t over. … There’ve been articles about people who have chosen MAiD for similar reasons and who have since died, including people with some of the diagnoses that I have. It’s just—people don’t understand. They don’t understand that this is taking the form of eugenics against disabled people, and poor people, and elderly people. And, like, MAiD, when it’s a genuine choice is one thing, I support that. But you can’t have that in *this* society. You can’t just, like, not provide people with what they need to live and then make it easier for them to die and call it a true choice, because it’s not. And it wasn’t for my friend. For me, it’s not something that I can just like get over, because it’s an injustice.

Hollis described how the emotional pain and grief over their friend’s death was inextricable from their disability justice perspective about the challenges of navigating the Canadian health care system as a person with a disability. Hollis’s disenfranchised grief is multilayered. They viewed their friend’s death as a state-sanctioned murder rather than a suicide, and they used emotionally charged language, including the term “eugenics,” to articulate their experience. They grieved the failure of the health care and social support systems to adequately care for people with disabilities, including themselves and their friends.

## Discussion

In this article, we describe how family members with complex MAiD bereavement experiences used pain descriptions in their bereavement stories. Physical pain was a moralizing agent that was used to justify why MAiD was or was not morally acceptable, whereas emotional pain described experiences of being dismissed and unacknowledged. These examples of dismissal constitute an epistemic and ethical harm called “testimonial injustice.” Testimonial injustice is a form of epistemic injustice that occurs when an individual’s speech acts are dismissed, disbelieved, or discredited either due to a prejudicial attitude toward the speaker’s social identity or because their perspective challenges a widely held belief.^28^ Emotional pain appeared in various contexts, including disagreements with family members and clinicians, in institutions, and within broader sociopolitical contexts. In all of the examples described, participants’ emotional pain represents disenfranchised grief.

Previous studies found that mental health challenges following a family member’s assisted death may be compounded by a lack of social support and experiences of stigma.^29^ Individuals may be judicious with whom they choose to disclose that their relative died from MAiD, which can lead to secrecy and social isolation.^8,11,13,15,16,30–32^ Hales and colleagues^32^ found that the burden of maintaining secrecy surrounding MAiD may lead to stress and anxiety, complicating the grieving process. Our study builds on these findings by further exploring the experience of emotional pain and disenfranchised grief among Canadians with complex MAiD bereavement experiences. Though several studies have noted the impact of stigma in the context of assisted death internationally, ours is the first study, to our knowledge, that explored the moral experiences of having one’s concerns be unacknowledged, dismissed, or invalidated.^8,11,13,15,16,29–32^

Based on our findings, we offer the following suggestions for improving care for family members with complex MAiD experiences and disenfranchised grief: First, clinicians should provide mental health resources such as information on grief counseling services and support groups. Second, there should be greater integration of health care ethics consultation services in cases where there is discordance between a patient and their family members. Health care ethicists can assist with mediating values-based family disagreements or conflicts and answer questions about legislative requirements. Third, there should be more effective training and education for mental health professionals on MAiD bereavement. Bereaved family members should not have to educate their therapists about MAiD to be provided with appropriate grief counseling and support. Fourth, suicide or death support groups may be helpful for individuals experiencing disenfranchised grief. Finally, clinicians involved in caring for patients accessing MAiD should be compassionate toward family members and validate their complex feelings and experiences.

## Limitations

The individuals who participated in this study identified themselves as white and of European descent. Many have achieved advanced degrees, and several are employed in law, research, or health care. Future research should explore the complex bereavement experiences of more racially and socioeconomically diverse individuals to understand how factors such as racism and access to social and material resources may intersect with complex MAiD bereavement.

## Conclusion

Experiences of physical and emotional pain leave a lasting impact on family members with complex MAiD bereavement. We conceptualize family members’ unacknowledged emotional pain as disenfranchised grief and argue that health care professionals should continue to improve care for family members following MAiD.

## Table 1.

**Table:** Participant demographic characteristics.

Characteristic	Qualitative study sample, *n* = 12
Gender, *n* (%)	
Female	8 (64)
Male	3 (25)
Nonbinary	1 (8)
Age range, *n* (%)	
25–34	1 (8)
35–44	0 (0)
45–54	3 (25)
55–64	2 (17)
65–74	4 (33)
75–84	2 (17)
Length of time in Canada, *n* (%)	
Born in Canada	10 (83)
Immigrated before age 10	2 (17)
Education, *n* (%)	
High school diploma	2 (17)
Undergraduate university degree	3 (25)
Master’s or professional degree	5 (42)
Doctoral degree	2 (17)
Relationship to person who had MAiD, *n* (%)	
Participant’s mother	2 (17)
Participant’s father	1 (8)
Participant’s husband	3 (25)
Participant’s sister	1 (8)
Participant’s brother	1 (8)
Participant’s close friend	4 (33)
Province where MAiD occurred, *n* (%)	
Ontario	7 (58)
British Columbia	3 (25)
Alberta	2 (17)
How long ago MAiD occurred, *n* (%)	
4–6 Months	4 (33)
7–12 Months	2 (17)
13–18 Months	2 (17)
19–24 Months	1 (8)
25–36 Months	1 (8)
36 + Months	2 (17)
